# Personalized organ donation directives: saving lives with PODDs

**DOI:** 10.1186/cc13872

**Published:** 2014-05-14

**Authors:** David Shaw

**Affiliations:** 1 Institute for Biomedical Ethics, University of Basel, Bernoullistrasse 28, 4056 Basel, Switzerland

## 

Despite various strategies that aim at persuading relatives to permit donation, the family veto of donation still constitutes a significant impediment to respecting potential donors’ wishes to have their deaths benefit others [1]. Personalised organ donation directives (PODDs) offer a potential solution to the problem of the family veto. PODDs would be cloud-based digital documents with video content that can be emailed to friends and family, and which also prompt the user to reconfirm organ intentions every year. Those who receive your PODD via email will receive a statement of your organ donation intent in text form with a digital signature, and a link that can be clicked in the event of your death, which will take them to the most recent version of your PODD (assuming that you do not want to send them yearly updates). PODDs also enable video messages to be recorded for families and friends, which will greatly increase the chances that organ donation intentions will be respected. While the main PODD text document is the same for all contacts, videos can be personalised for different groups so that families can be asked to respect donation wishes and friends asked to ensure that families do so. The video component is optional but this element is key to making the PODD an effective persuasive tool. Box 1 illustrates the 10-minute process of setting up a PODD.

PODDs offer several advantages to those wishing to donate their organs after death. The most important facet is their personalised nature. Rather than being shown a tattered organ donor card by a nurse, or simply being told that their relative was on the register, or faintly half-remembering a decades-old conversation, family members will vividly remember a specific video statement that they received; if they have forgotten it, they can be shown it again. Furthermore, PODDs are much more contemporaneous than alternative methods of stating organ intentions: if relatives question whether any stated intentions are current, they can be shown the annual reconfirmations and any updates to the PODD. Additionally, if any relatives are still reluctant, other relatives and friends who are perhaps more up-to-date with the donor’s intentions will have their resolve to prevent use of a veto strengthened by the PODD.

Ideally, access to PODDs could be integrated into national donor registers, with a link from the register to the PODD document and video(s), so that doctors can facilitate family access to PODDs for those who do not use email or when disputes arise. Another advantage of PODDs is that they can be used to stop any family disagreements about what the deceased would have wanted. Essentially, PODDs are extremely powerful persuasive tools that virtually guarantee that families will not veto donation by addressing the key reasons given by families for refusing donation [2]. Widespread adoption of PODDs by those already on the register would probably encourage those who had not seriously thought about donation to sign up, particularly if PODD videos (by both living and deceased people) were also used as part of promotional campaigns aimed at increasing donation. PODDs would make donation decisions both personal and perpetual, and have the potential to eradicate the family veto.

### Box 1. Setting up a PODD

1. Go to PODD website.

2. Fill in organ directive form.

3. Record short video.

4. Enter email addresses of recipients.

5. Choose reconfirmation frequency.

6. Sign and submit.

## Abbreviations

PODD: Personalized organ donation directive.

## Competing interests

The author declares that he has no competing interests.
